# Bone marrow MSCs in MDS: contribution towards dysfunctional hematopoiesis and potential targets for disease response to hypomethylating therapy

**DOI:** 10.1038/s41375-018-0310-y

**Published:** 2018-12-21

**Authors:** Zhiyong Poon, Niraja Dighe, Subhashree S. Venkatesan, Alice M. S. Cheung, Xiubo Fan, Sudipto Bari, Monalisa Hota, Sujoy Ghosh, William Y. K. Hwang

**Affiliations:** 1 Department of Hematology, Singapore General Hospital, Singapore, Singapore; 2 Department of Clinical Translational Research, Singapore General Hospital, Singapore, Singapore; 3 Cardiovascular & Metabolic Disorders and Centre for Computational Biology, Duke-NUS Medical School, Singapore, Singapore; 4 National Cancer Center, Singapore, Singapore; 5 Cancer & Stem Cell Biology, Duke-NUS Medical School, Singapore, Singapore

**Keywords:** Cancer stem cells, Chemotherapy

## Abstract

The study of myelodysplastic syndromes (MDS) in murine models has now indicated the possible involvement of the bone marrow microenvironment in the generation of dysplastic hematopoietic cells. However, there is scant work on patient samples and the role of hypomethylating agents on the bone marrow stromal cells of MDS patients is unclear. We show that human MDS-MSCs exhibit phenotypic, transcriptomic and epigenetic abnormalities. Stimuli provided by MDS-MSCs impaired the growth and function of healthy HSPCs, which is further sustained autonomously in HSPCs for significant periods of time resulting in a failure for active hematopoietic engraftment across primary and secondary transplant recipients (chimerism: 0.34–91% vs 2.78%, engraftment frequencies: at 0.06 ± 0.02 vs full engraftment for MDS-MSC vs healthy groups, respectively). Hypomethylation of MDS-MSCs improved overall engraftment in most of the MDS-MSC groups tested (2/7 with *p* *<* 0.01, 3/7 with *p* *<* 0.05 and 2/7 with no significant difference). MDS-MSCs that fail to respond to hypomethylating therapy are associated with patients with rapid adverse disease transformation and this further suggests that MDS-MSCs may be an integral part of disease progression and have prognostic value as well as potential as a therapeutic target.

## Introduction

Mesenchymal stromal cells (MSCs) are a principal component of the BM hematopoietic niche that regulate the activities of closely associated hematopoietic cells to facilitate either normal blood cell development or the survival and propagation of blast cells [1, 2]. To perform the latter, MSCs acquire various uncharacteristically dysfunctional properties that generate conditions for reinforcing cancer development. Compared to age-matched healthy controls, dysplastic MSCs derived from patients with pre-leukemic disorders (MDS) or AML have an altered secretome and surface protein profile that confers pro-survival benefits, immunomodulation or chemotherapy resistance to leukemic cells [3–9]. These studies suggest that through long periods of exposure to a stimulatory MDS milieu, healthy stromal cells are epigenetically reprogrammed to function in cooperation with leukemic cells and propagate the disease as a whole.

The realization of this new symbiotic relationship in the pathogenesis of hematopoietic malignancies raises the other possibility that dysplastic stromal cells may also initiate or unmask latent potential for cancer formation in healthy HSPCs. This phenomenon, if true, may also account for clinical observations of secondary or donor derived leukemia [10]. The number of investigations in this area is rapidly growing in recent years with several hallmark studies verifying in murine cancer models that genetic perturbations (Dicer1 deletions, Wnt/β-catenin mutations, etc) in healthy BM-MSCs can be the sole orchestrating force for the onset of hematopoietic abnormalities that resemble leukemia [11–13]. A recent study has also demonstrated that multiplex gene editing to confer leukemic drivers in healthy human HSPCs is insufficient for the development of leukemia after transplantation in mice [14], supporting the need for a dysplastic stroma in disease initiation. Changes in the state of MSCs as a result of disease can potentially have far-reaching effects on the hematopoietic system, but direct translation of these observations for human cancers is still speculative. Less work has been done to document how dysplastic stromal cells (eg. MSCs) from patients may impact the course of an emerging hematopoietic malignancy. However, some of the identified MSC genetic pathways (Wnt/β-catenin, proinflammatory genes, Jagged-1, miR-155) that are implicated in murine cancer models have been correlated with human clinical outcomes [12, 13, 15–17].

The new appreciation of the role that dysplastic BM stromal cells play in initiating or propagating certain hematopoietic disorders (eg. MDS) as a ‘disease unit’ may underscore the importance of having therapy that is also targeted against dysplastic MSCs or stromal cells to achieve better outcomes. Treatment offered for MDS is risk-adapted according to the IPSS [18–20]. Patients with lower-risk (LR) MDS are mainly given supportive care, but patients with higher-risk (HR) MDS receive AZA as frontline and subsequently, stem cell transplantation where appropriate. Herein, we examined the role of MSCs in different MDS-risk groups and subsequently evaluated the effects of hypomethylating agents on their biology and function as hematopoietic regulators. We find that exposure of healthy hematopoietic cells to MDS stromal cells (particularly in the HR group) has long-term deleterious effects on healthy CD34^+^ HSPCs. Hypomethylating treatment of MDS-MSCs rescued hematopoiesis in the majority of HR-MDS experimental groups; these observations suggests that a possible mechanism of action of these drugs is through normalization of the BM microenviroment and advocates the need for development of more efficient stromal targeting moieties for such diseases. There is a growing body of work in this area [3, 21–25], and our study extends this knowledge through examinations of patient samples in vivo and the finding that MDS-MSC responses to AZA may have certain prognostic value.

## Materials and Methods

(Please see Supplementary Information for more details for each section)

### Human samples

Cryopreserved BM MDS samples (*n* = 21 patients) were obtained from the hematology repository at the Singapore General Hospital (SGH). Healthy MSCs (*n* = 6 donors) were derived from healthy donors at SGH after consent. BM CD34^+^ cells (*n* = 2 donors) were purchased from Lonza. This study is approved under the SingHealth CIRB 2014/664/F. Informed consent was obtained from all patients and donors.

### In vitro assays

#### MSC differentiation

MSCs were differentiated to osteogenic and adipogenic lineages for quantification of calcium and fat forming potential, respectively.

#### Co-culture experiments

Healthy or MDS-MSCs (with or without AZA treatment) were plated at a near confluent density of 2.0 × 10^4^ cells/cm^2^ in D10 medium. 24 h later, healthy CD34^+^ HSPCs were seeded in contact with the MSC feeder layer at a density of 2.0 × 10^3^ cells/cm^2^ in hematopoietic media (see Supplemental Information) and cultured for up to 14 days with replacement of media after 7 days. On the last day, CD34^+^ HSPCs were flow sorted on the BD ARIA and subjected to subsequent experimentation.

#### Treatment of MDS-MSCs with azacitidine (AZA)

Subconfluent MSCs at P1–P2 were treated with 1 µM AZA (Sigma-Aldrich) for up to 48 h. We determined the optimal working concentration of AZA using a series of qPCR measurements of representative gene expression (Supplementary Figure 2). After exposure to AZA, cells were washed with PBS and replaced with drug free media. The drug-treated MSCs were then maintained in AZA free media for further experimentation.

### DNA methylation and RNA-seq analysis

Methylation data was imported into Partek Genomic Suite 7.0 for analysis. Unsupervised PCA clustering and supervised ANOVA analysis was performed. A significance value of *p* *<* 0.001 and a difference in beta value of <−0.1 or >0.1 was used to identify important differentially methylated probes. All RNA-seq data was processed on Partek Flow. Raw data was aligned to hg19 using STAR 2.5.3a. Partek GSA were used to detect differential expressed genes between treated vs not treatment groups. The default setting for GSA is LIMMA [26]. *p* < 0.05 were considered significant. The list of significant genes was used for pathway enrichment analysis based on KEGG human pathway database.

### In vivo experimentation

Eight- to 12-weeks-old female NSG (NOD-*scid* IL2Rgamma^null^) mice (Jackson Laboratory) mice were sublethally irradiated (240 cGy) 24 h before transplantation, randomized and subsequently injected intravenously with FACS sorted CD34^+^ HSPCs at a dose of 1.0 × 10^7^ cells/kg (Fig. 5a). BM was analyzed at week 8 to determine engraftment and multilineage differentiation of human cells. Human CD34^+^ HSPCs from primary engrafted mice were FACS sorted and serially transplanted into secondary recipients for another 8 weeks. A minimum sample size of *n* > 5 was used. All animal experiments were performed under an approved protocol (2014/SHS/1005).

## Results

### Patient characteristics

Tissue samples from 21 MDS patients and 6 healthy donors were used in this study. The characteristics of our study group are given in Table 1. 11 patients that were classified as lower-risk (LR) MDS patients had a lower marrow blast count (<5%, range 0–3%) while 10 were classified as higher-risk (HR) MDS patients and had a higher marrow blast count (>5%, 6–13%). Histological assessment of BM cellularity showed a higher incidence of hypercellularity in the marrow of HR-MDS patients (Table 1). The median age of the patients was 46 (range: 35–49), 76 (range: 33–84) and 61.5 (33–77) for healthy, LR and HR groups, respectively. The incidence of cytogenetic abnormalities in hematopoietic cells and overall mortality due to leukemia was higher in the HR-risk group (Cytogenetics: 0.60(HR) vs 0.45(LR), Mortality: 0.50(HR) vs 0.36(LR)). Complex HSPC karyotype abnormalities were detected in 2/11 and 5/10 of LR- and HR-MDS patients, respectively. LR-MDS patients presented with refractory cytopenias (RARS, RCMD and RCUD) while HR-MDS patients presented with RAEB1 and RAEB2. FISH analysis with a MDS panel (LSIs: EGR1, D7S486, D8Z2 and D20S108) was used to corroborate findings.

**Table 1:** Diagnosis and characteristics of MDS study cohort.

Patient ID	Risk Classification	BM Histopathology	Hematopoietic Karyotype	Mesenchymal Karyotype	Hematopoietic FISH panel	Blasts	Classification
150	Low	Not performed	Not performed	—	Not performed	0%	RARS
285	Low	Normocellular marrow for patient’s age (20-30%) Stromal changes: Hemoserous changes seen	46,XX[20]	—	Not performed	1%	UNCLASSIFIED
290	Low	Normocellular marrow for patient’s age (20-30%) Stromal changes: None	46,XY[20]	—	Negative	0%	RARS
364	Low	Hypercellular marrow for patient’s age (40-50%) Stromal changes: Storage iron is noted focally and may be increased	45,XY,-18,der(20)t(18;20)(q11.2;q13.2)[18]/ 46,idem, + der(20)t(18;20)(q11.2;q13.2)[2]	—	Not performed	1%	RARS
378	Low	Hypercellular marrow for patient’s age (85%) Stromal changes: None	44,XY,add(1)(p13),add(4)(q21),-5,der(6)t(1;6)(p13;q27), -7,add(12)(p11.2)[cp6]/46,idem,-add(12)(p11.2), + 12, + mar1[cp2]/45,XY,add(1)(p13),add(3)(p12),-5, der(6)t(1;16)(p13;q27),-7,-21, + mar2, + mar3, + mar4[6]/ 45,XY,add(1)(p13),-5,der(6)t(1;6)(p13;q27),add(7)(q22), -10, + mar5[cp4]/(46,XY[2]	—	Not performed	2%	RCMD
419	Low	Hypercellular marrow for patient’s age (60-70%) Stromal changes: None	46,XY[20]	—	Not performed	0%	RARS
482	Low	Hypocellular marrow for patient’s age (25%) Stromal changes: None Reticulin: 1/ + 4, normal	46,XY,i(17)(q10)[19]/46,XY[1]	—	Negative	5%	UNCLASSIFIED
582	Low	Hypercellular marrow for patient’s age (80-90%) Stromal changes: Haemoserous exudate Reticulin: 1 + /4, normal, focal, fine fibre pattern	46,XY[20]	—	Not performed	0%	RARS
739	Low	Hypercellular marrow for patient’s age (50%) Stromal changes: None Reticulin: 1 + /4, normal	46,XY[20]	—	Negative	0%	RCUD
801	Low	Hypercellular marrow for patient’s age (80-90%) Stromal changes: Focal fibrosis with increased storage iron Reticulin: 1 + /4, normal	46,X,i(X)(p10)[8]/47,idem, + i(X)(p10)[cp2]/46,XX[10]	—	Negative	0%	RARS
819	Low	Normocellular marrow for patient’s age (20-30%) Stromal changes: None Reticulin: 1 + /4, normal	47,XY, + 8[cp13]/46,XY[7]	—	Positive	3%	RCUD
261	High	Hypercellular marrow for patient’s age (60%) Stromal changes: None Reticulin: 1 + /3, increased fibrosis	46,XX,del(1)(p32p34),add(2)(p13),del(5)(q13q35),add(20) (q11.2)[3]/45,idem,-7,del(12)(p11.2)[cp14]/46,XX[2]	45,XX,-22[7]/46,idem, + mar[7]/46,XX[6]	Negative	13%	RAEB2
305	High	Hypercellular marrow for patient’s age (80-90%) Stromal changes: None Reticulin: 1 + /4, normal	46,XX[20]	46,XX[20]	Not performed	12%	RAEB2
325	High	Normocellular marrow for patient’s age (80-90%) Stromal changes: None Reticulin: 2 + /4, moderate increase fibrosis	45~46,XX,add(5)(q11.2),-7,-12,der(21)t(12:21)(q11:p13), + r, + mar[cp16]/46,XX[4]	46,XX[20]	Not performed	10%	RAEB1
400	High	Hypercellular marrow for patient’s age (80-90%) Stromal changes: Haemoserous exudate Reticulin: 1 + /4, normal	46,XY[20]	46,XY[20]	Not performed	13%	RAEB2
455	High	Hypercellular marrow for patient’s age (70%) Stromal changes: None Reticulin: 2 + /4, normal	46,XY, + 1,der(1;15)(q10;q10)[3]/46,XY[17]	46,XY[20]	Negative	7%	RAEB1
613	High	Hypercellular marrow for patient’s age (70%) Stromal changes: There is mild increase in reticulin, featuring a diffuse network of fine reticulin fibres. No stromal collagenosis or necrosis. Reticulin: 2/4	46,XY, + 1,der(1;15)(q10;q10)[2]/46,XY,dup(1)(q21q31)[3] /46,XY[15]	46,XY[20]	Not performed	8%	RAEB1
627	High	Hypercellular marrow for patient’s age (60%) Stromal changes: None Reticulin: 0 + /4, normal	46,XX[20]	46,XX[20]	Negative	8%	RAEB1
717	High	Hypercellular marrow for patient’s age (40-50%) Stromal changes: Lysis and edema Reticulin: 1 + /4, normal	46,XY[20]	46,XY[20]	Negative	8%	RAEB1
768	High	Hypercellular marrow for patient’s age (70-80%) Stromal changes: None Reticulin: 1 + /4, normal	48,XY, + 1,del(5)(q22q35), + 11[20]	46,XY[9]	Positive	6%	RAEB1
775	High	Hypercellular marrow for patient’s age (90%) Stromal changes: None Reticulin: 3 + /4, increased	44~46,XY,add(3)(q12),-5,-6,der(7;11)(p10;q10), + 11,-17, + 19,-20,-22, + 4~5mar[cp3]/45~47,XY,-5, + 6, der(6)del(6)(p23p25)add(6)(q13),-7,-11, + 17, add(17)(p11.2), + 19,del(19)(p13.3)x2,-20, + 22, add(22)(p11.2),add(22)(p11.2), + 1~5mar[cp14]/46,XY[2]	46,XY[20]	Positive	6%	RAEB1

### MDS-MSC characteristics

**Fig. 1:**
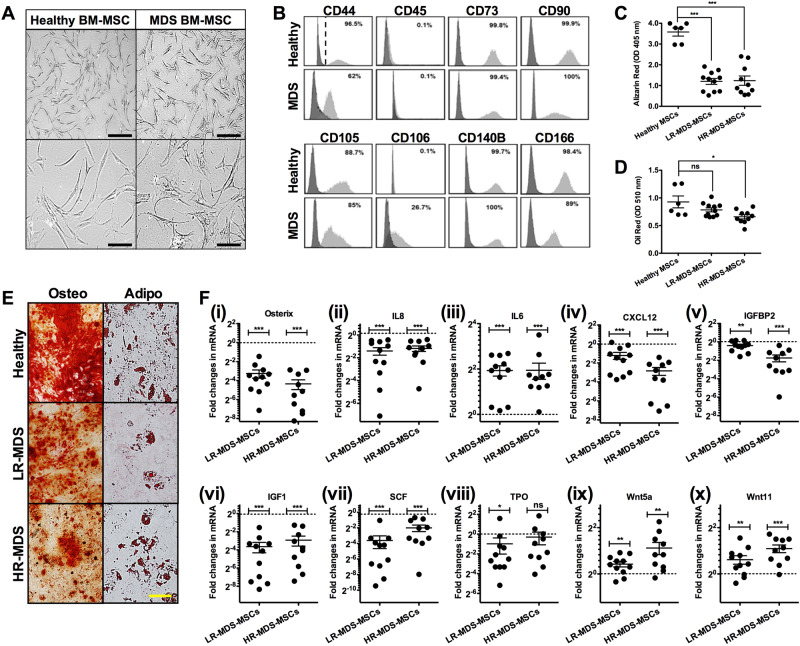
Characteristics of MDS-MSCs. **a** A comparison of the morphology of healthy and MDS-MSCs. In contrast to healthy MSCs, MDS-MSCs generally have non-spindle shaped morphology. Scale bars = 100 μm (top bars) and 40 μm (bottom bars). **b** Representative surface immunophenotype analysis of healthy (n = 6) vs MDS-MSCs (n = 21) using flow cytometry which show expression of CD73, CD90, CD105, CD166, CD140B and no expression of CD45 in all samples (healthy or MDS). However, expression of CD44 (*n* = 21; range 26–90%) and CD106 (*n* = 21; range: 10–26.7%) was varied across MDS samples. Dark histogram = Isotype control, Gray histogram = respective surface marker. **c**, **d** Quantification of osteogenic (**c**) and adipogenic (**d**) differentiation potential in MSCs using Alazarin Red and Oil Red, respectively, which is then extracted and spectroscopically measured (See Methods Section). Each data point represents a donor or patient sample. Both LR-MDS-MSCs (*n* = 11) and HR-MDS-MSCs (*n* = 10) had significantly reduced osteogenic differentiation potential against healthy MSCs (*n* = 6 healthy donors shown in graph). Adipogenic differentiation potentials were also reduced but not significantly in LR-MDS-MSCs but significantly in HR-MDS-MSCs. **e** Representative images of osteogenic and adipogenic differentiation in different MSCs. Scale bar = 40 μm. **f** (i–viii)) qPCR analysis of MDS-MSCs (HR- and LR-MDS, P1) for expression of genes related to MSC function or hematopoietic support. Each data point represents a patient sample. Compared to healthy MSCs, LR- and HR-MDS-MSCs showed the following fold changes, respectively - Osterix: 0.11 ± 0.03 and 0.05 ± 0.02, IL8: 0.38 ± 0.09 and 0.44 ± 0.07, IL6: 3.82 ± 0.60 and 3.85 ± 1.00, CXCL12: 0.43 ± 0.11 and 0.14 ± 0.04, IGFBP2: 0.76 ± 0.08 and 0.30 ± 0.08, IGF1: 0.08 ± 0.03 and 0.13 ± 0.05, SCF: 0.08 ± 0.04 and 0.25 ± 0.07, TPO: 0.51 ± 0.26 and 0.81 ± 0.34, Wnt5a: 1.40 ± 0.10 and 2.18 ± 0.39, Wnt11: 1.51 ± 0.21 and 2.13 ± 0.24. The expression levels of these genes against each patient sample normalized to a healthy donor controls are given in Supplementary Figure 3. *p *<* 0.05, **p *<* 0.01, ***p *<* 0.001, ns not significant. Non-paired student’s *t* test was performed. All data represented as mean ± SEM

Primary MDS patient and healthy donor samples were processed in a similar workflow to derive MSCs for analysis and experimentation at low passage (P) numbers (Supplementary Figure 1A); this process reduces the impact of ex vivo culture on the original biology of MSCs. Tissue samples were obtained at the point of initial diagnosis or disease relapse. Compared to healthy controls, MDS-MSCs generally present non-spindle shaped morphology and the colonies also appear disorganized as well as smaller in size (Fig. 1a). FACS analysis showed that MDS-MSCs express similar levels of MSC surface markers such as CD73, CD90, CD105, CD166 and CD140B (*n* = 21) and are negative for CD45 (*n* = 21). Expression of CD44 (*n* = 21; range 20–90%) and CD106 (*n* = 21; range: 8–26.7%) was varied across MDS samples (Fig. 1b), which may be a response to an inflammatory microenvironment [27]. At P0, healthy MSCs could be expanded from ~7.5 × 10^4^ cells to an average of 6.5 × 10^5^ cells in 7 days (doubling time: 2.22 ± 0.04 days; *n* = 6)). In contrast, the doubling time of MDS-MSCs was slower at 5.53 ± 0.32 days (*n* = 10; *p* *<* 0.01) and 6.82 ± 1.44 days (*n* = 11; *p* *<* 0.05) for HR- and LR-MDS-MSCs, respectively (Supplementary Figure 1B). All MDS-MSCs showed reduced osteogenic differentiation potential (*p* *<* 0.001 for both HR- and LR-MDS groups, Fig. 1c, e). The reduction in adipogenic differentiation potential was not significant in LR-MDS-MSCs but was significant in HR-MDS-MSCs (*p* *<* 0.05, Fig. 1d, e). Cytogenetic analyses performed on the group of HR-MDS-MSCs showed only 1 of 10 (10%) samples with abnormalities (Table 1) [28].

We performed qPCR analysis of MDS-MSCs (*n* = 21, P1) for a range of genes that have been shown to be important to MSC differentiation or hematopoiesis. Compared to healthy controls, both HR- and LR-MDS-MSCs showed significant (*p* < 0.001) reduction in the expression of osteogenic markers such as Osterix and IL8 (Fig. 1f (i–iii)). Proinflammatory cytokine IL6 (Figure F(iv)) was significantly increased in both MDS groups compared to healthy controls (*p* < 0.001) [29]. The expression levels of hematopoietic factors such as CXCL12, IGFBP2, IGF1, SCF and TPO (Fig. 1f (v–viii)) were also reduced (*p* < 0.001, except for TPO). In agreement with recent studies [13, 21], we further found heightened levels of Wnt5a and Wnt11 expression in all MDS-MSCs but more significantly so in HR-MDS-MSCs (*p* < 0.01, Fig. 1f (ix) and 1F(x)).

### Epigenetic dysregulation in MDS-MSCs

**Fig. 2:**
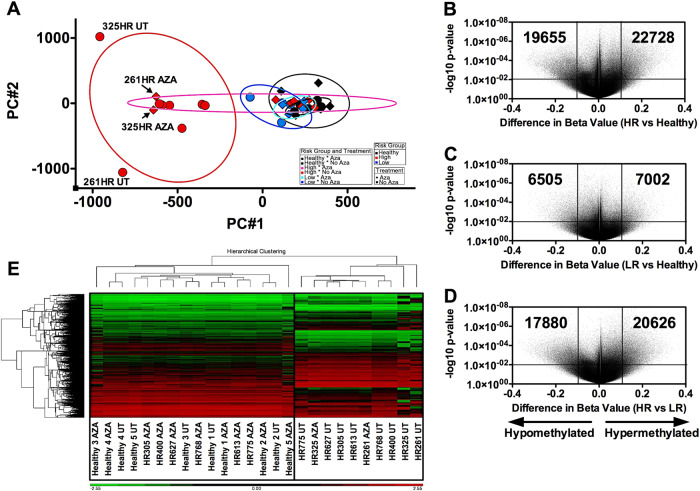
Epigenetic dysregulation in MDS-MSCs. **a** Unsupervised PCA analysis of a set of 5 healthy (black), 8HR-MDS (261HR, 305HR, 325HR, 400HR, 613HR, 627HR, 768HR, 775HR, red) and 4 LR-MDS (364LR, 378LR, 482LR and 739LR, blue) MSC samples, before (circles, UT) and after (diamonds, AZA) in vitro treatment with AZA reveal distinct groupings. Untreated HR-MDS-MSCs were most epigenetically distinct from untreated healthy or LR-MDS-MSCs. AZA treatment was effective in normalizing the epigenetic profile of all LR-MDS-MSCs (4/4) and most HR-MDS-MSCs (6/8, partial response: 261HR and 325HR). **b**–**d** Volcano plots of the significance (*p* value) against differences between methylation levels of individual CpG loci of different sample groups. MDS-MSCs are more hypermethylated than hypomethylated (22728 vs. 19655 and 7002 vs 6505 hypermethylated loci vs. hypomethylated loci for HR- and LR-MDS-MSCs vs. healthy MSCs, respectively). **e** Hierarchical clustering of samples using CpG loci that are significantly differentially methylated distinguishes HR-MDS-MSCs vs healthy MSCs, but is not as effective for LR-MDS-MSCs vs healthy MSCs (Supplementary Figure 4A). Additionally, these groupings also reveal that 261HR and 325HR remain more epigenetically similar to untreated counterparts than healthy MSCs, indicating partial response to AZA

In view of the low levels of MDS-MSC cytogenetic abnormalities from our cohort and other reported studies [28], we used the Infinium Methylation EPIC array to interrogate the methylation patterns of CpG dinucleotides in a set of 5 healthy, 8HR-MDS (261HR, 305HR, 325 h, 400HR, 613HR, 627HR, 768HR, 775HR) and 4 LR-MDS (364LR, 378LR, 482LR and 739LR) MSC samples, before and after in vitro treatment with AZA. We had excluded certain MDS-MSCs from this analysis due to lack of availability. Principal component analysis (PCA) based unsupervised clustering revealed distinct groupings of our samples (Fig. 2a). Untreated HR-MDS-MSCs were most epigenetically distinct from untreated healthy or LR-MDS-MSCs. Following AZA treatment, 6/8HR-MDS-MSC samples clustered more closely with untreated healthy samples. There were changes in the epigenetic profile of 261HR and 325HR MSCs following AZA treatment, but not sufficient enough to cause closer association with healthy controls. We next performed a supervised analysis of the methylation profiles using ANOVA on all untreated samples. Volcano plots of the significance (*p* value) against differences between methylation levels of individual CpG loci of different sample groups are given in Fig. 2b–d. Compared to healthy MSCs, the overall methylation profiles of HR-MDS-MSCs are more aberrant than LR-MDS-MSCs (Fig. 2b–d). MDS-MSCs are more hypermethylated than hypomethylated (22728 vs 19655 and 7002 vs 6505 hypermethylated loci vs hypomethylated loci for HR- and LR-MDS-MSCs vs healthy MSCs, respectively). Hierarchical clustering of samples using CpG loci that are significantly differentially methylated (Fig. 2b–d) showed that untreated HR-MDS-MSCs are distinct from healthy MSCs (Fig. 2e). However, this set of differentially methylated CpGs was not as efficient in distinguishing untreated LR-MDS-MSCs from healthy controls (Supplementary Figure 4A). The set of differentially methylated CpGs identified in Fig. 2b–d included genes that we assayed for expression in Fig. 1f, such as IGFBP2, CXCL12 etc, which show clear groupings via their methylome between healthy and MDS samples (Supplementary Figure 4B and 4C, respectively). In both unsupervised and supervised analysis, AZA treatments of 261HR and 325HR did not establish a methylation profile that is closer to healthy MSCs, suggesting that these samples may be resistant to hypomethylating therapy.

### Degeneration of healthy HSPCs in a MDS microenvironment

**Fig. 3:**
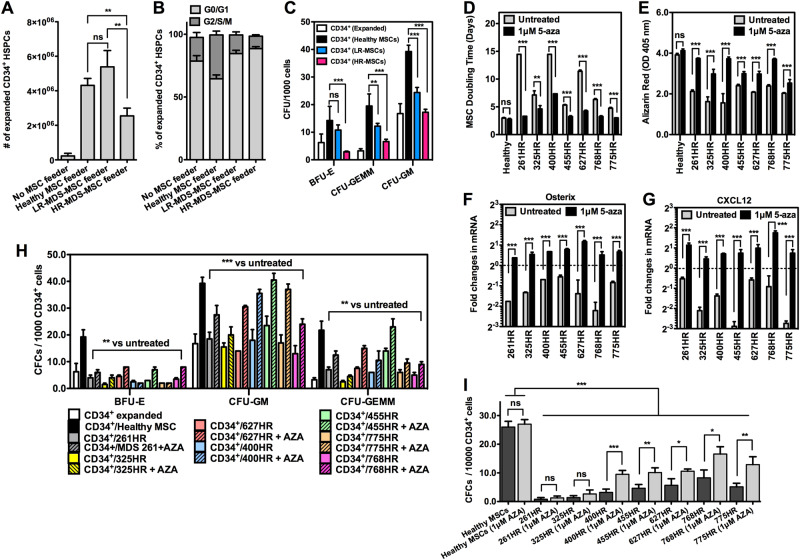
Dysplastic MDS-MSCs impairs healthy HSPCs. **a** There was a significant reduction in the number of CD34^+^ HSPCs from co-cultures with HR-MDS-MSCs (n = 9, 2.55 ± 0.45 × 10^6^) compared to healthy MSCs (n = 6, 4.31 ± 0.40 × 10^6^, *p* *<* 0.01) or LR-MDS-MSCs (n = 8, 5.39 ± 0.95 × 10^6^, *p* *<* 0.01) but no significant difference between LR-MDS-MSC and healthy MSC groups. **b** Cell cycle analysis at day 7 shows the smallest percentage of cycling HSPCs when co-cultured on MDS-MSC stroma, particularly HR-MDS-MSCs. The percentages of cells in G2/S/M are 18.88 ± 3.90, 35.01 ± 3.02, 14.88 ± 2.39 and 9.88 ± 1.92 for no feeder cultures, healthy MSC, LR-MDS-MSC and HR-MDS-MSC co-cultures, respectively. **c** Hematopoietic CFC potential of healthy CD34^+^ HSPCs after brief expansion under co-culture conditions with healthy (n = 6), HR-MDS- (n = 8) or LR-MDS-MSCs (n = 8). Attenuation of differentiation potential was most pronounced in HSPCs after co-culture with the HR-MDS-MSC group. Frequencies for CFU-BFU were 2.9 ± 0.3 vs. 14.3 ± 5.1, those for CFU-GM were 17.2 ± 1.2 vs. 39.3 ± 2.3 and those for CFU-GEMM were 6.6 ± 0.9 vs. 19.5 ± 4.4 for the HR-MDS-MSC (*n* = 9) vs. healthy MSC (*n* = 6) groups, respectively. Individual CFC counts for each sample are given in Supplementary Figure 5B. These results demonstrate phenotypic changes in HSPCs after exposure to HR-MDS-MSCs. **d** The average doubling time of MSCs before and after AZA treatments at P0–P1. For healthy MSCs, treatment did not result in appreciable improvements to proliferative capacities (~1.1 fold increase), but for the experimental set of HR-MDS-MSCs (*n* = 7), treatment resulted in 1.6–4.4 fold increases in the rate of proliferation, *p* *<* 0.001. **e** Quantification of osteogenic differentiation potential in MSCs (P0 - P1) before and after AZA treatments. No significant improvements in osteogenic differentiation potential was observed in healthy treatment MSCs (1.1 fold increase), but 1.2–2.4 fold improvements to osteogenic differentiation potentials were observed in treated HR-MDS-MSCs (*n* = 7). **f**, **g** qPCR analysis of MDS-MSCs (*n* = 7, P1) for expression of Osterix and CXCL12 before and after AZA treatments. Representative data normalized to a healthy control is shown. After treatment, gene expression of Osterix and CXCL12 significantly increased. Similar trends were observed with IL8 and IGF1 gene expression (Supplementary Figure 6). **h** Hematopoietic CFC potential of CD34^+^ HSPCs following co-culture on treated vs untreated HR-MDS-MSCs further showed significant improvements (*p* *<* 0.001 or *p* *<* 0.01) in the number of CFU-GM (~1.9 × ) and GFU-GEMM (~1.9 × ) compared to co-culture with untreated MDS-MSCs. These data show that hypomethylating drugs such as AZA may also target dysplastic MDS stromal cells and contribute indirectly to the overall restoration of active hematopoiesis. **i**) The LTC-IC CFC output following a period of co-culture for 5 weeks using different feeder layers. CFCs from all HR-MDS-MSCs (untreated or AZA treated) were significantly lower than healthy co-cultures (*p* *<* 0.001). However, AZA treatments of HR-MDS-MSCs were able to partially restore LTC-IC supporting capabilities (*p* *<* 0.05), except for 261HR and 325HR (no significant improvements). *p *<* 0.05, **p *<* 0.01, ***p *<* 0.001, ns not significant. Non-paired student’s *t* test was performed. All data represented as mean ± SEM

We first compared in vitro co-cultures of healthy adult BM CD34^+^ HSPCs (Lonza) in direct contact with various MSC types (HR-, LR-MDS groups as well as healthy). After 14 days, co-culture with healthy (*n* = 6) or MDS-MSCs (*n* = 17) resulted in comparable numbers of CD45^+^ hematopoietic cells (1.22 ± 0.1 × 10^7^ and 1.46 ± 0.21 × 10^7^ cells, respectively; *p* *=* 0.16) (Supplementary Figure 5A). When CD34^+^ HSPCs were enumerated, we found a significant reduction in the number of HSPCs from HR-MDS-MSC co-cultures (n = 9, 2.55 ± 0.45 × 10^6^) compared to healthy MSCs (*n* = 6, 4.31 ± 0.40 × 10^6^, *p* *<* 0.01) or LR-MDS-MSCs (*n* = 8, 5.39 ± 0.95 × 10^6^, *p* *<* 0.01) but no significant difference between LR-MDS-MSC and healthy MSC groups (Fig. 3a). Cell cycle analysis of expanded CD34^+^ HSPCs at day 7 shows that the HR-MDS-MSC group had the lowest number of cells in G2/S/M phase (9.87 ± 3.1 %) compared to the healthy MSC group (35.03 ± 3.02 %, *p* *<* 0.001) (Fig. 3b). These data indicate that MDS-MSCs, and especially HR-MDS-MSCs, support HSPC expansion poorly. The hematopoietic CFC potential of co-cultured CD34^+^ HSPCs was subsequently evaluated to determine if short-term expansion with MDS-MSCs affects hematopoietic lineages (Fig. 3c). CD34^+^ HSPCs from all MDS-MSC co-cultures showed a reduced CFC potential compared to HSPCs from healthy MSC co-cultures, but this attenuation was most pronounced in HSPCs after co-culture with the HR-MDS-MSC group. Frequencies for CFU-BFU were 2.9 ± 0.3 vs 14.3 ± 5.1, those for CFU-GM were 17.2 ± 1.2 vs. 39.3 ± 2.3 and those for CFU-GEMM were 6.6 ± 0.9 vs. 19.5 ± 4.4 for the HR-MDS-MSC (*n* = 9) vs. healthy MSC (*n* = 6) groups, respectively. Individual CFC counts for each sample are given in Supplementary Figure 5B. These results demonstrate phenotypic changes in healthy HSPCs after exposure to HR-MDS-MSCs.

Given the close functional relationship between hematopoietic and mesenchymal cells within BM niches, we considered if hypomethylating agents such as AZA could indirectly affect MDS hematopoietic cells by acting through MDS-MSCs. We first examined if the abnormal characteristics of MDS-MSCs (Fig. 1) could be reversed with hypomethylating therapy, focusing on a set of 7HR-MDS-MSCs (261HR, 325HR, 400HR, 455HR, 627HR, 768HR and 775HR, Fig. 3d–g). After treatment, all 7HR-MDS-MSCs showed significantly increased proliferative capacities (1.6–4.4 fold increase, *p* *<* 0.001, Fig. 3d) and osteogeneic differentiation potentials (*p* *<* 0.001, Fig. 3e) in comparison to same patient untreated MDS-MSCs. We also observed significant increases in gene expression of Osterix (*p* *<* 0.001, Fig. 3f) and IL8 (*p* *<* 0.001, Supplementary Figure 6A), corroborating with improved osteogenic potential (Fig. 3e). The expression of CXCL12 (*p* *<* 0.001, Fig. 3g) and IGF1 (*p* *<* 0.001, Supplementary Figure 6B) were also improved, which suggest a restoration of their ability to support hematopoiesis.

Subsequently, we performed short-term co-culture expansions using AZA treated HR-MDS-MSCs and healthy donor-derived CD34^+^ HPSCs as before. The frequency of all types of hematopoietic CFC in healthy CD34^+^ HSPCs following co-culture on AZA treated HR-MDS-MSCs also increased significantly (average fold increase for CFU-GM: 1.8 ± 0.1, CFU-GEMM: 1.9 ± 0.1, BFU-E: 1.7 ± 0.2) compared to those co-cultured with untreated MDS-MSCs (Fig. 3h). In order to determine the effect on primitive hematopoietic progenitors after exposure to untreated vs. AZA-treated MDS stroma, we performed bulk culture LTC-IC assays using HR-MDS-MSCs as feeder layers. After maintaining healthy CD34^+^ HSPCs for 5 weeks, the number of LTC-IC derived CFCs from all HR-MDS-MSCs (untreated or AZA treated) were significantly lower than healthy co-cultures (*p* *<* 0.001). However, AZA treatments of HR-MDS-MSCs were able to partially restore LTC-IC supporting capabilities (*p* *<* 0.05), except for HR261 and HR325. These data show that hypomethylating drugs such as AZA may also target dysplastic MDS stromal cells and contribute indirectly to the overall restoration of active hematopoiesis. However, this is not efficacious in all HR-MDS-MSCs; samples HR261 and HR325, which were obtained from patients in < 1 year before disease transformation leading to death were only partially responsive to AZA therapy. This reduced effect of AZA was also observed in our epigenetic analysis (Fig. 2e).

### RNA-seq analysis of healthy CD34 + HSPCs co-cultured with different MDS-MSC systems

**Fig. 4:**
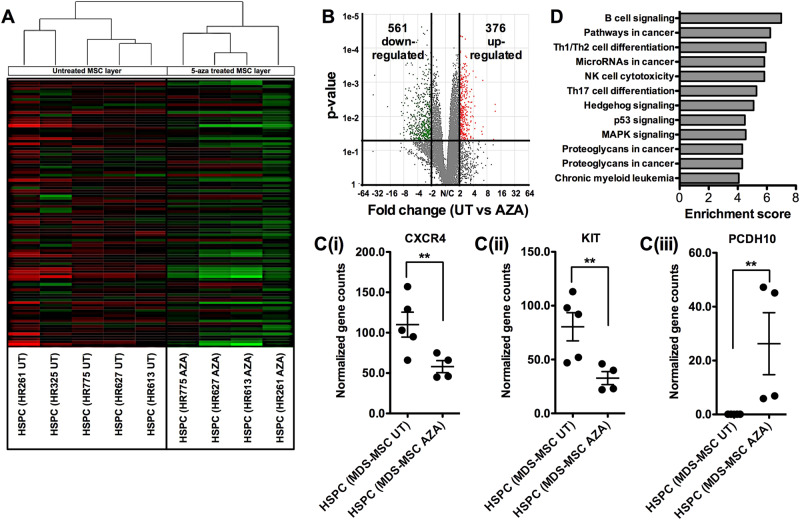
RNA-seq analysis of healthy CD34^+^ HSPCs after co-culture with different MDS-MSCs. **a** Hierarchical clustering of HSPCs co-cultured with MDS-MSCs (HR261, HR325, HR613, HR627 and HR775). HDS-MSCs are treated with AZA (AZA) or untreated (UT). HSPCs from HR325 AZA co-cultures were insufficient for this analysis. Clustering of differentially expressed genes show distinct groupings between HSPCs that were co-cultured on UT vs AZA MDS-MSCs. HSPCs co-cultured with AZA treated HR261 MDS-MSCs were less similar to HSPCs from other treated systems, similar to previous observations (Fig. 3). **b** There are more down-regulated genes in HSPCs from UT vs AZA MDS-MSCs. Using a *p* < 0.05 and FC > 2, 561 vs 376 genes were down- vs up-regulated, respectively. **c** Normalized gene counts from gene specific analysis (Partek Flow) of CXCR4 (i), KIT (ii) and PCDH10 (iii). CXCR4 and KIT are commonly seen up-regulated in leukemic blasts and PCDH10 is a tumor suppressor that is silenced by hypermethylation. **d** KEGG pathway analysis of differentially expressed genes in HSPCs after co-culture with UT vs. AZA MDS-MSCs indicates activation of cancer/aberrant differentiation signaling networks in healthy HSPCs after exposure to dysplastic MDS-MSCs. *p *<* 0.05, **p *<* 0.01, ***p < 0.001, ns not significant. Non-paired student’s *t* test was performed. All data represented as mean ± SEM

We next performed RNA-seq on CD34^+^ sorted HSPCs from different co-cultures systems (HR261, HR325, HR613, HR627 and HR775) for both AZA and untreated stroma. HSPCs from co-cultures with untreated MDS-MSCs were distinct in gene expression compared to their treated counterparts (Fig. 4a). However, similar to previous observations (Figs. 2 and 3), HSPCs co-cultured with AZA treated HR261 MDS-MSCs were less similar to HSPCs from other treated systems. Overall, there were more significantly down-regulated genes in HSPCs from untreated vs treated co-culture systems (561 vs 376, *p* < 0.05, FC > 2, Fig. 4b). Differentially expressed genes include CXCR4 and KIT (Figures C(i) and C(ii), respectively), which were up-regulated, as well as PCDH10 (Figure C(iii)), which was down-regulated in untreated co-culture systems. Up-regulation of CXCR4 and KIT is associated with leukemic blasts and PCDH10 is a common tumor suppressor that is also epigenetically silenced in hematopoietic malignancies [30–34]. KEGG analysis of the differentially expressed genes indicates activation of pathways associated with cancer, chronic myeloid leukemia, Th1, Th2 and Th17 differentiation, as well as p53 and hedgehog signaling (Fig. 4d). Together, these data further lend support to potential roles that MDS-MSCs play towards leukemogenesis from healthy HSPCs and also demonstrate that reversing dysplastic MDS-stroma may contribute towards overall patient recovery.

### Exposure to dysfunctional MDS-MSCs has long-term deleterious effects on healthy CD34^+^ HSPCs in transplantation models

**Fig. 5:**
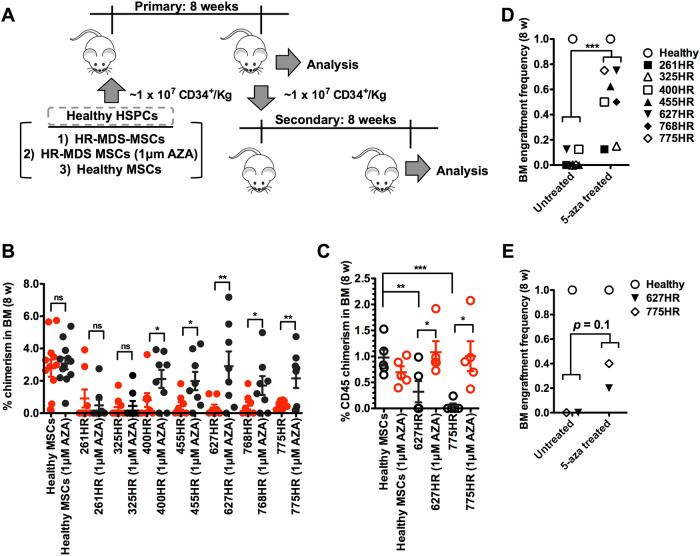
Epigenetic targeting of MDS-MSCs reverses long-term deleterious effects on healthy CD34^+^ HSPCs. **a** Schematic of transplantation experiments with FACS sorted human BM CD34^+^ HSPCs from different MSC co-cultures. **b** Percentage chimerism of human CD45^+^ cells in the BM of irradiated (240 cGy) NSG mice 8 weeks after transplantation. Human CD34^+^ HSPC dose was fixed among all groups tested at ~1 × 10^7^ cells/kg and only HSPCs were injected. At least 8 mice were used per experimental group and each data point represents chimerism in one mouse. Values for untreated and AZA experimental groups are – healthy: 2.79 ± 0.53 and 3.07 ± 0.36, 261HR: 0.91 ± 0.55 and 0.96 ± 0.63, 325HR: 0.37 ± 0.22 and 0.45 ± 0.31; 400 h: 0.81 ± 0.43 and 2.12 ± 0.56, 455HR: 0.45 ± 0.23 and 1.99 ± 0.56, 627 h: 0.34 ± 0.19 and 2.90 ± 0.91, 768HR: 0.46 ± 0.23 and 1.71 ± 0.58, 775HR: 0.49 ± 0.10 and 2.15 ± 0.60, respectively. Overall, chimerism of human cells was significantly lower after in co-culture groups with HR-MDS-MSCs (*p* < 0.01 for each MDS group vs healthy MSCs). No differences were observed in the general marrow architecture of the two experimental groups (Supplementary Figure 7). Pre-treatment of MSCs with AZA led to significant improvements in BM chimerism for 5/7HR-MDS-MSC groups (400HR (2.6 × ), 455 h(4.5 × ), 627HR (76 × ), 768HR(3.7 × ) and 775HR (4.3 × )). No appreciable improvements to BM chimerism was observed in the AZA-treated healthy MSC, 261HR and 325HR groups. **c** In secondary recipient mice (*n* = 5), we found negligible chimerism of human CD45^+^ cells in the BM of HR627 and HR775 groups compared a secondarily transplanted healthy HSPC groups. However, higher levels of chimerism were found in AZA-treated HR627 and HR775 groups (*p* *<* 0.05). **d** In the primary transplant the engraftment frequencies (threshold of 0.5% human CD45^+^ cells in the BM) for untreated vs AZA experimental groups are – healthy: 1 and 1, 261HR: 0 and 0.13, 325HR: 0 and 0.15, 400 h: 0.13 and 0.5, 455HR: 0 and 0.63, 627HR: 0 and 0.75, 768HR: 0 and 0.5, 775HR: 0 and 0.75, respectively. **e** In secondary transplanted mice, the engraftment frequencies (threshold of 0.5% human CD45^+^ cells in the BM (see Supplementary Figure 8 for representative plots used to determine minimum threshold of 0.5%), for untreated vs. AZA experimental groups are—healthy: 1 and 1, 627HR: 0 and 0.20, 775HR: 0 and 0.40, respectively. Note that engraftment efficiencies were trending higher although not quite mathematically significant (*p* *=* 0.1). Together, these results demonstrate significant effects on both progenitors and HSCs after exposure to MDS stroma. The treatment of MDS stromal cells with hypomethylating agents such as AZA has the potential to correct this disorder. *p *<* 0.05, **p *<* 0.01, ***p *<* 0.001, ns not significant. Non-paired student’s *t* test was performed. All data represented as mean ± SEM

CD34^+^ HSPCs recovered from 10 to 14 days of co-culture with 1) HR-MDS-MSCs, 2) AZA treated HR-MDS-MSCs and 3) healthy MSCs were intravenously injected into sublethally irradiated NSG recipients at similar dose levels. 8 weeks post-primary transplant, human CD34^+^ HSPCs from whole BM was isolated and secondarily transplanted for evaluation after another 8 weeks (Fig. 5a). Human hematopoietic repopulation was severely diminished after transplantation of HSPCs co-cultured with HR-MDS-MSCs (from 7 different HR-MDS-MSC samples) vs healthy MSCs. In the primary recipient, the average human chimerism achieved by xenografted HSPCs in the BM ranged from 0.34 to 0.91% in the HR-MDS-MSC expanded groups compared to 2.78% in the healthy MSC group (Fig. 5b). The frequencies of engraftment, determined by a threshold of 0.5% human CD45^+^ cells in the BM, was also significantly lower at 0.06 ± 0.02 for HR-MDS-MSC groups (*n* = 8 mice, Fig. 5d), compared to full engraftment in healthy MSC groups (*n* = 12 mice). Pre-treatment of HR-MDS-MSCs before co-culture improved chimerism human cells, but this effect was not evident in all HR-MDS-MSC groups tested (2/7 with *p* *<* 0.01, 3/7 with *p* *<* 0.05 and 2/7 with no significant difference, Fig. 5b). Overall, engraftment frequencies were improved to 0.49 ± 0.10 after AZA pre-treatment (*n* = 8, *p* *<* 0.001, Fig. 5d). Further analysis of human cells in the BM at 8 weeks showed no significant differences in lineage composition compared to control groups, indicating that MDS stroma exposure did not skew the differentiation potential of HSPCs (Supplementary Figure 9).

We transplanted pooled human HSPCs from 2 primary experimental arms (HR627 and HR775) into secondary irradiated NSG mice (n = 5) to investigate if the effects of dysplastic MDS-MSCs also extend to the more primitive hematopoietic cells. Human CD34^+^ HSPCs were lower in prevalence in the BM of HR627 (0.26 ± 0.07%) and HR775 (0.12 ± 0.05%) groups compared to the healthy MSC (1.3 ± 0.39%) group (*p* *<* 0.05). 8 weeks after secondary transplantation, we found negligible chimerism and engraftment (0.5% threshold) of human CD45^+^ cells in the BM of HR627 and HR775 groups (average chimerism at 0.32 and 0.05 for HR627 and HR775, respectively, engraftment frequencies at 0 for both, Fig. 5c, e). Higher levels of chimerism were found in AZA treated HR627 and HR775 groups (*p* *<* 0.05). Engraftment efficiency was also improved from 0 (untreated) to 0.30 ± 0.10 (treated) (not mathematically significant but trending higher, *p* *=* 0.1). These results demonstrate significant long-term effects on active hematopoietic reconstitution (both progenitors and primitive HSCs) after exposure to MDS stroma that may be partially corrected by treatment of stromal cells with hypomethylating agents such as AZA.

## Discussion

Our data demonstrates that MSCs derived from MDS patients are dysfunctional and have the ability to induce abnormalities in healthy HSPCs. MDS-MSCs have irregular morphologies, form disorganized colonies and have significantly reduced differentiation capacities. The significant loss of osteogenic potential in MDS-MSCs suggests a change in MSC biology to a phenotype that has a reduced capacity for supporting healthy HSPCs [35]. Hematopoiesis is sustained in BM niches by MSCs through CXCR4/CXCL12 signaling [36, 37] as well as through a variety of secreted factors such as SCF, TPO, IGF1, etc [3, 37–40]; these factors were all found to be generally dysregulated in gene expression studies (Fig. 1). Increase in IL6 may reflect a proinflammatory phenotype in the MDS BM. Co-culture of healthy HSPCs with HR-MDS-MSCs reduced hematopoietic expansion of CD34^+^ cells and CFC potential, particularly in the GM and GEMM lineages. In transplantation experiments that tested their fitness for engraftment, we observed significant attenuation of engraftment potential in both primary and secondary recipients of healthy CD34^+^ HSPCs that were exposed to HR-MDS-MSCs.

Changes in the epigenome of MDS-MSCs via stimulation by a MDS milieu are potentially important drivers of these abnormalities. We observed clear changes to the epigenome, but a high level of karyotypic and genetic abnormalities were not as evident in MDS-MSCs [28, 41] (Table 1). Subsequently, we determined that treatment with hypomethylating therapy (AZA) significantly reverses abnormalities in MDS-MSC properties (osteogenic differentiation, proliferation, gene expression, etc) and also leads to significant improvements in their ability to support hematopoietic cells for in vivo engraftment (Figs. 3 and 5). It is interesting to note that AZA treatments of 261- and 325HR-MDS-MSC were unsuccessful in rescuing its ability to maintain LTC-ICs (Fig. 3i) and in vivo engraftment potential (Fig. 5b); this failure of therapy may be associated to further cytogenetic or molecular genetic abnormalities (Table 1).

Importantly, the observation of impaired hematopoietic regeneration in healthy CD34^+^ HSPCs after exposure to MDS-MSCs is noteworthy because it shows that hematopoietic abnormality can be induced and be sustained autonomously in HSPCs for significant periods of time (16 weeks across two recipient mice) by a MDS stroma. The observed effects using MDS-MSCs that were expanded in vitro is also striking, as it establishes that in spite of the current view that alterations to MSCs in MDS marrow are most often a secondary adaptation of the disease [3, 6, 23], these transformed MDS-MSCs are self-replicative to propagate biological abnormalities to their progenies and can potentially exert a long-lasting effect in vivo.

In correlating our experimental results with clinical findings, we first note that both 261HR- and 325HR-MDS-MSC samples were taken at the onset of disease relapse and approximately 6–12 months before the patient passed away due to transformation of MDS to acute leukemia. At this late stage of the disease, it is possible that these MSCs may already harbor abnormalities that are so extensive that would render them partially responsive to the effects of hypomethylating therapy (Fig. 2). Patients contributing samples 305HR, 400HR, 455HR and 613HR (between 2011 and 2012) are still alive at this present moment, have slow or no disease progression and have responded well to AZA; patient 627 h (sample collected in 2012) responded well to AZA for a period of time but subsequently stopped responding and is currently being treated for AML progression since 2016; patients 717HR, 768HR and 775HR (samples collected between one to two years before death) also showed progression to AML. Thus, it is tempting to speculate that MDS-MSCs may also have some prognostic value as the degree of impairment of these cells may also suggest severity of disease at the time of sample collection. These observations are in support of the notion that hematopoietic disorders like MDS are propagated as a disease ‘unit’ [6].

Taken together, these findings have important repercussions on future investigations of the pathogenesis of MDS and approach to treatment. First, the notion that some aspects of human MDS can be MSC-driven leads to a paradigm shift in the manner in which we view and characterize them. While the involvement of MSCs in chemoresistance mechanisms and the survival of blast cells is well established, the possibility of de novo initiation of hematopoietic disorders via the stromal environment is still largely unexplored [42]. Second, such studies could provide the basis for introduction of new cancer drugs that target the stromal microenvironment and the means for identifying patient populations that may benefit from such therapy. These new therapies can be implemented to work in synergy with current front-line hematopoietic-targeting drugs for putting the MDS into remission and/or used as a pre-transplant conditioning therapy to eliminate residual dysplastic MSCs and reduce relapse risk following transplant.

## Supplementary information

Supplemental Information (Clean)
