# Correction: Bone marrow MSCs in MDS: contribution towards dysfunctional hematopoiesis and potential targets for disease response to hypomethylating therapy

**DOI:** 10.1038/s41375-019-0406-z

**Published:** 2019-02-20

**Authors:** Zhiyong Poon, Niraja Dighe, Subhashree S. Venkatesan, Alice M. S. Cheung, Xiubo Fan, Sudipto Bari, Monalisa Hota, Sujoy Ghosh, William Y. K. Hwang

**Affiliations:** 1 Department of Hematology, Singapore General Hospital, Singapore, Singapore; 2 Department of Clinical Translational Research, Singapore General Hospital, Singapore, Singapore; 3 Cardiovascular & Metabolic Disorders and Centre for Computational Biology, Duke-NUS Medical School, Singapore, Singapore; 4 National Cancer Center, Singapore, Singapore; 5 Cancer & Stem Cell Biology, Duke-NUS Medical School, Singapore, Singapore

**Correction to:** **Leukemia**;

10.1038/s41375-018-0310-y;

published online: 21 December 2018.

In the original version of this article there was a mistake in the spelling of the author Sujoy Ghosh, originally spelt Sujoy Gosh. This has now been corrected in both the PDF and HTML versions of the article.

