# Supplementary material for: Bone marrow MSCs in MDS: contribution towards dysfunctional hematopoiesis and potential targets for disease response to hypomethylating therapy
Source: Leukemia. 2018 Dec 21;33(6):1487–500. doi: 10.1038/s41375-018-0310-y (PMC6756222; doi:10.1038/s41375-018-0310-y)
Supplement: Supplementary file 1 — Supplemental Information (Clean) [file 41375_2018_310_MOESM1_ESM.docx]

**Bone marrow MSCs in MDS: contribution towards dysfunctional hematopoiesis and potential targets for disease response to hypomethylating therapy**

**Supplementary Materials and Methods**

**Cells and Culture**

For MSCs, the culture reagents used were: Dulbecco’s Modified Eagle’s Medium (DMEM) - low glucose (Gibco) supplemented with 10% fetal bovine serum (FBS) (Hyclone) and 50 U/ml penicillin and streptomycin (Gibco), subsequently referred to as D10 media. For HSPCs, the culture reagents used were: StemSpan SFEM (STEMCELL Technologies) supplemented with cytokine cocktail of 100 ng/mL human stem cell factor (SCF) (PeproTech), 100 ng/mL human thrombopoietin (TPO) (PeproTech) and 50 ng/mL human FLT-3 Ligand (FLT-3) (PeproTech).

*Derivation of MSCs:* To derive MSCs, 2.0 - 3.0 × 10^5^ cells/cm^2^ of BM derived mononuclear cells were inoculated in D10 media that was changed completely after 3 days and subsequently replaced twice a week until confluence and cryopreserved for experiments at P1. MDS patient derived mononuclear cells (1.0 × 10^6^ – 1.0 × 10^7^ cells/ml) were also inoculated in D10 media in a T75 flask. Non-adherent cells were removed after 3 days with subsequent media change twice a week. MDS-MSCs were further expanded, frozen down and all experiments were carried out using MSCs derived from P1 - P2 (Supplementary Figure 1).

*Isolation of MDS CD34^+^ HSPCs:* CD34^+^ HSPCs were isolated from BM MDS mononuclear cells using the CD34^+^ MicroBead Kit and magnetic-activated cell sorting (MACS) (Miltenyi Biotec).

***Methylation and RNA-seq***

*DNA methylation:* DNA was extracted from cells using the DNeasy Blood and Tissue Kit (Qiagen) and methylation profiles were measured using the Infinium Human MethylationEPIC BeadChip (Illumina). The microarray service was provided by Macrogen Inc. (South Korea). Raw DNA methylation data was processed by first applying quality control (“minfi” R-package), followed by normalization with the beta-mixture quantile normalization (BMIQ) method (“wateRmelon” R-package). Finally, probe-wise differential methylation analysis (“limma” R-package) was conducted to filter out all probes that had a less than 10% average methylation rate and less than 0.106455 (M-value) fold change between untreated and 5-aza treated samples. (NCBI GEO accession: GSE119617)

*RNA-seq:* Paired end raw data fastq files were imported into Partek Flow 7.0. The sequences were aligned to hg19 using STAR version 2.5.3a default parameters. Transcript abundance was determined using Partek E/M algorithm based on Ensembl Transcripts release 75^1^. Raw read count were generated and normalized for sequence depth –RPM, a small offset of 0.0001 were added to each value to deal with 0s. Low expression genes are indistinguishable from noise, so genes with total raw counts less than or equal 10 were removed during quantification. After normalization, another filter was performed so that only genes with geometric average greater than or equal to 1 are used for the downstream analysis. Partek GSA were used to detect differential expressed genes between treated vs not treatment groups. The default setting for GSA is LIMMA^2^. *p* < 0.05 were considered significant. The list of significant genes was used for pathway enrichment analysis based on KEGG human pathway database. (NCBI GEO accession: GSE119916)

***In Vitro* Assays**

*Flow cytometry analysis:* Surface immunophenotype analyses were performed using FACS for the following markers: CD34 (AC136), CD45 (REA747), CD73 (REA804), CD90 (REA897), CD105 (REA794), CD166 (REA442), CD44 (REA690), CD146 (REA773), CD140B (REA363) and CD106 (REA269) (antibodies from BD and Miltenyi).

*MSC Proliferation:* The proliferative capacities of MDS-MSCs were assessed by determining the doubling times of MDS-MSCs that were plated at 1.0 x 10^3^ cells/cm^2^ in D10 media for a week.

*qPCR studies:* RNA was extracted using the RNeasy Mini Kit (Qiagen) and converted to cDNA using the Omniscript Reverse Transcriptase Kit (Qiagen). Quantitative PCR was performed in duplicate on the CFX96 TouchTM Real Time PCR Detection System using the SYBR Green Master Mix (Bio Rad). Primer sequences are given in Supplementary Table 1. mRNA expression levels of genes were normalized to healthy controls and GAPDH. Fold change was calculated by the 2-ddCT method.

*MSC differentiation:* For osteogenic induction, MSCs were plated at 2.0 × 10^4^ cells/cm^2^ and cultured in osteogenic differentiation media (DMEM supplemented with 10.0 mM β-glycerophosphate, 1.0 × 10^-5^ mM dexamethasone, and 0.2 mM ascorbic acid) for 14 days, with media change twice a week. Extracellular accumulation of calcium was measured by Alizarin Red S staining. The stained monolayer was extracted with 10% acetic acid (v/v) followed by colorimetric quantification at 405 nm. For adipogenic induction, MSCs were plated at 2.0 × 10^4^ cells/cm^2^ and cultured in adipogenic differentiation medium (DMEM supplemented with 5 µg/ml insulin, 1.0 × 10^-6^ M dexamethasone, and 0.6 × 10^-4^ M indomethacin) for 2 weeks, with media change twice per week. To detect cytoplasmic lipid accumulation, cultures were fixed in 4% paraformaldehyde for 20 min and stained with 0.3% Oil Red O in 60% isopropanol for 15 mins. Oil Red O content in samples was quantified by extraction with 100% isopropanol for 5 min followed by colorimetric quantification at 510 nm.

*LTC-IC assay:* Long-term culture-initiating cell assays (LTC-IC) in bulk culture were carried out using different MSCs as feeder cells. MSCs were plated confluent on collagen coated 12 well plates, treated with mitomycin C (200 µg/mL) and replaced with myelocult H5100 media supplemented with fresh hydrocortisone (10 µM hydrocortisone 21-hemisuccinate, Sigma). These feeders were used to maintain 1.0 × 10^4^ healthy CD34^+^ HSPCs for 5 weeks, with weekly 50% media change. CFCs were enumerated as indicated below.

*CFC assay:* From co-cultures, patient or donor samples, 1.0 × 10^3^ FACS sorted CD34^+^ HSPCs were seeded in StemMACS HSC-CFU media with EPO (Miltenyi Biotec) and performed in duplicate. CFU colonies (BFU-E, CFU-GM and CFU-GEMM) were scored after 14 days under a microscope.

*RNA-seq:* RNA was extracted from sorted CD34^+^ HSPCs using the RNeasy Mini Kit (Qiagen).

*Cell cycle analysis:* HSPCs were isolated and washed, fixed (paraformaldehyde), permeabilized (triton X) and stained with propidium iodide (PI) for analysis of different cell cycle stages on the flow cytometer. Approximately 10,000 cells were analyzed with excitation at 488 nm and emission at 617 nm.

***In Vivo* Assays**

*Murine bone marrow harvest and derivation of CD34^+^ HSPCs:* Transplanted mice were euthanized with CO_2_ and both femurs were removed intact, washed and dried with a sterile paper towel. The prepared femur bones were cut into two sections at the middle and flushed with a 21G needle with sterile DMEM. The flushed marrow was spun down (500 g for 5 min) and resuspended in blood lysis buffer (Sigma Aldrich) to remove RBCs (10 min incubation). The treated cells were then passed through a 70 μm filter, using a 5 ml syringe plunger to push all cells through. These cells were then pelleted and stained with antibodies for relevant analysis on the flow cytometer. For determination of chimerism, hCD45-APC (REA747) and mCD45-FITC (REA737) were used. Chimerism was determined as: 100 × (hCD45/(hCD45 + mCD45)) %. Engraftment frequency was determined as the percentage of transplanted mice that had a minimum threshold of 0.5 % human CD45^+^ cells in the BM (see Supplementary Figure 8 for representative plots used to determine minimum threshold of 0.5%). hCD19 (REA675), hCD33 (REA775) and hCD3 (BW264/56) antibodies were used for determination of B, myeloid and T cells, respectively. For isolation of human CD34^+^ cells for secondary transplantation, hCD34^+^ (AC136) hCD38^-^ (REA671) hLin- cells were isolated by flow cytometry. Experimental arms were not blinded to the investigators. All antibodies were obtained from Miltenyi Biotech or BD Biosciences.

**Data analysis**

*Statistics:* Unless otherwise stated, non-paired student t-tests were performed for all data comparisons, with **p* < 0.05, ***p* < 0.01, ****p* < 0.001, ns = Not significant. All data represented as mean ± SEM.

**Supplementary Figures and Captions**

**
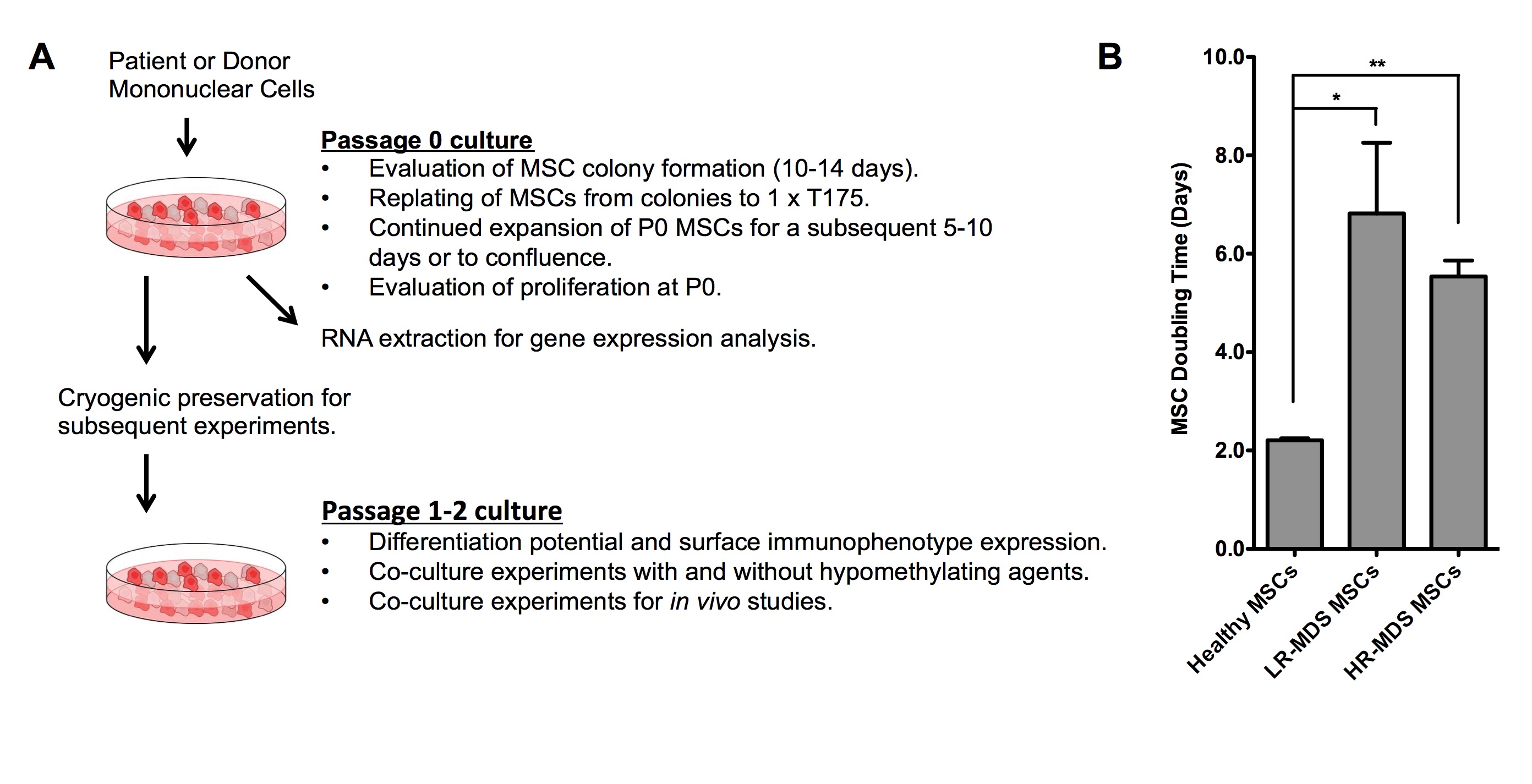
**

**Supplementary Figure 1.** **A)** Schematic showing sample processing from donors and patients. All samples were processed in a similar workflow to derive MSCs for analysis and experimentation at low passage (P) numbers (up to P1-2). **B)** The average doubling time of MSCs at P0 for healthy MSCs, HR-MDS-MSCs and LR-MDS-MSCs were 2.22 ± 0.04 days (n = 6), 5.53 ± 0.32 days (n = 10) and 6.82 ± 1.44 days (n = 11), respectively. The possibility of cell death during culture was not a factor considered in these calculations.

**
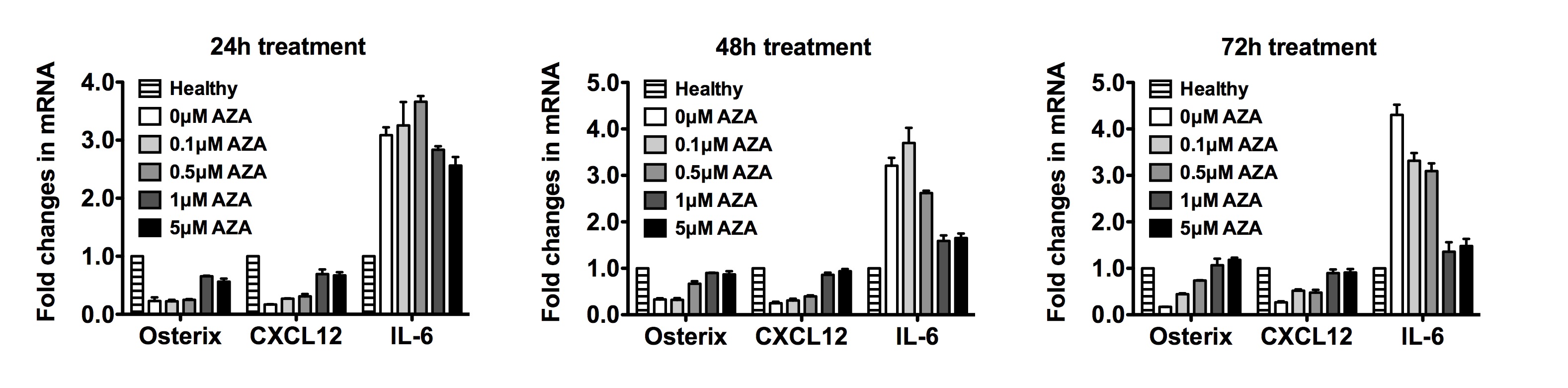
**

**Supplementary Figure 2.** Determining optimal dosing of AZA using representative gene expression analysis in MDS-MSCs. We tested down- (Osterix, CXCL12) and up- (IL-6) regulated genes in MDS-MSCs (HR305) before and after treatment with different AZA concentrations and timepoints. These results show that the optimal treatment is at a dose of 1.0 μM for 48h.

**
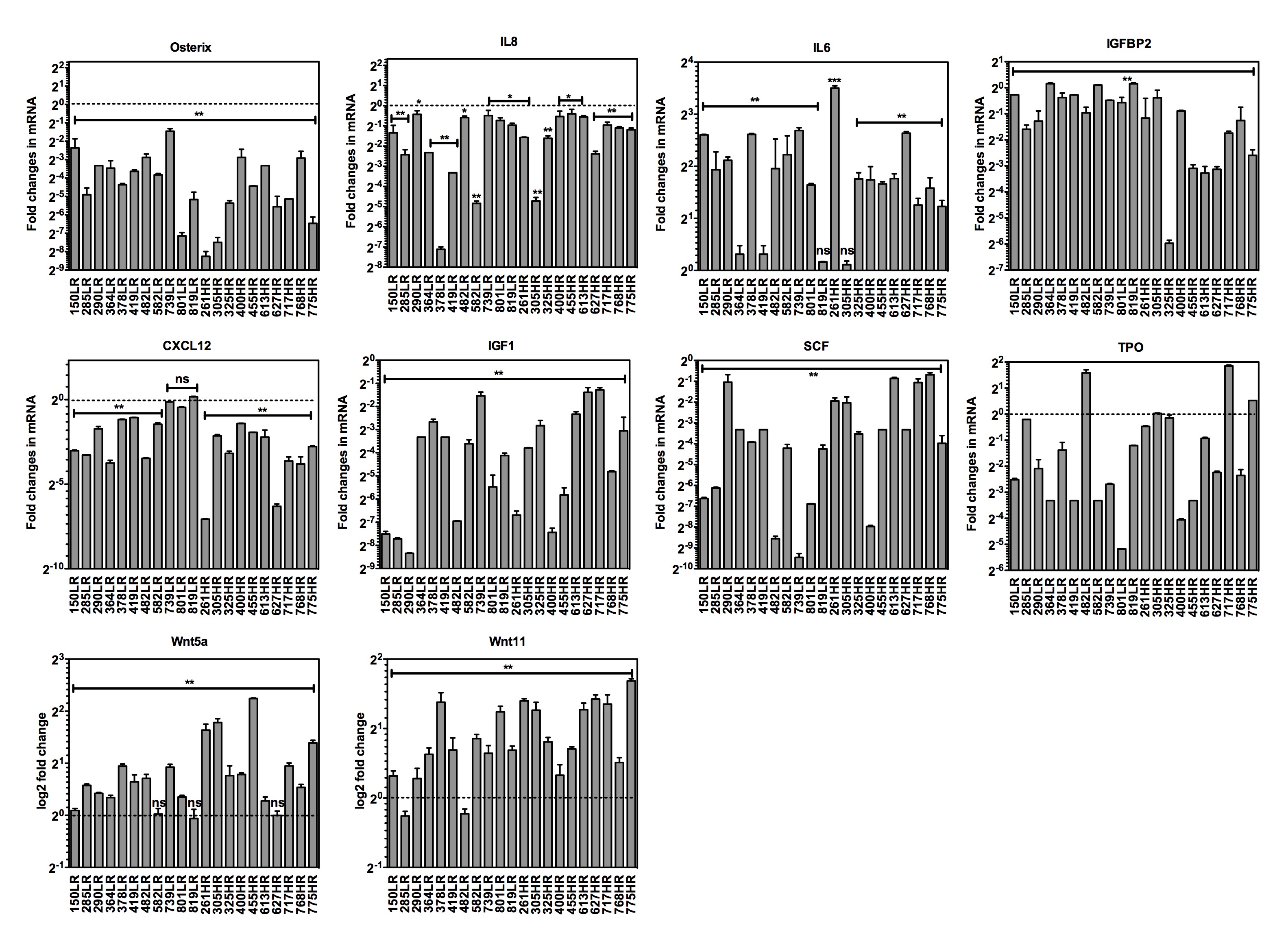
**

**Supplementary Figure 3.** qPCR analysis of MDS-MSCs (n = 21, P1) for expression of genes related to MSC function or hematopoietic support. Individual patient sample data was normalized to a healthy control. **p* < 0.05, ***p* < 0.01, ****p* < 0.001, ns = Not significant. Non-paired student’s t-test was performed. All data represented as mean ± SEM.


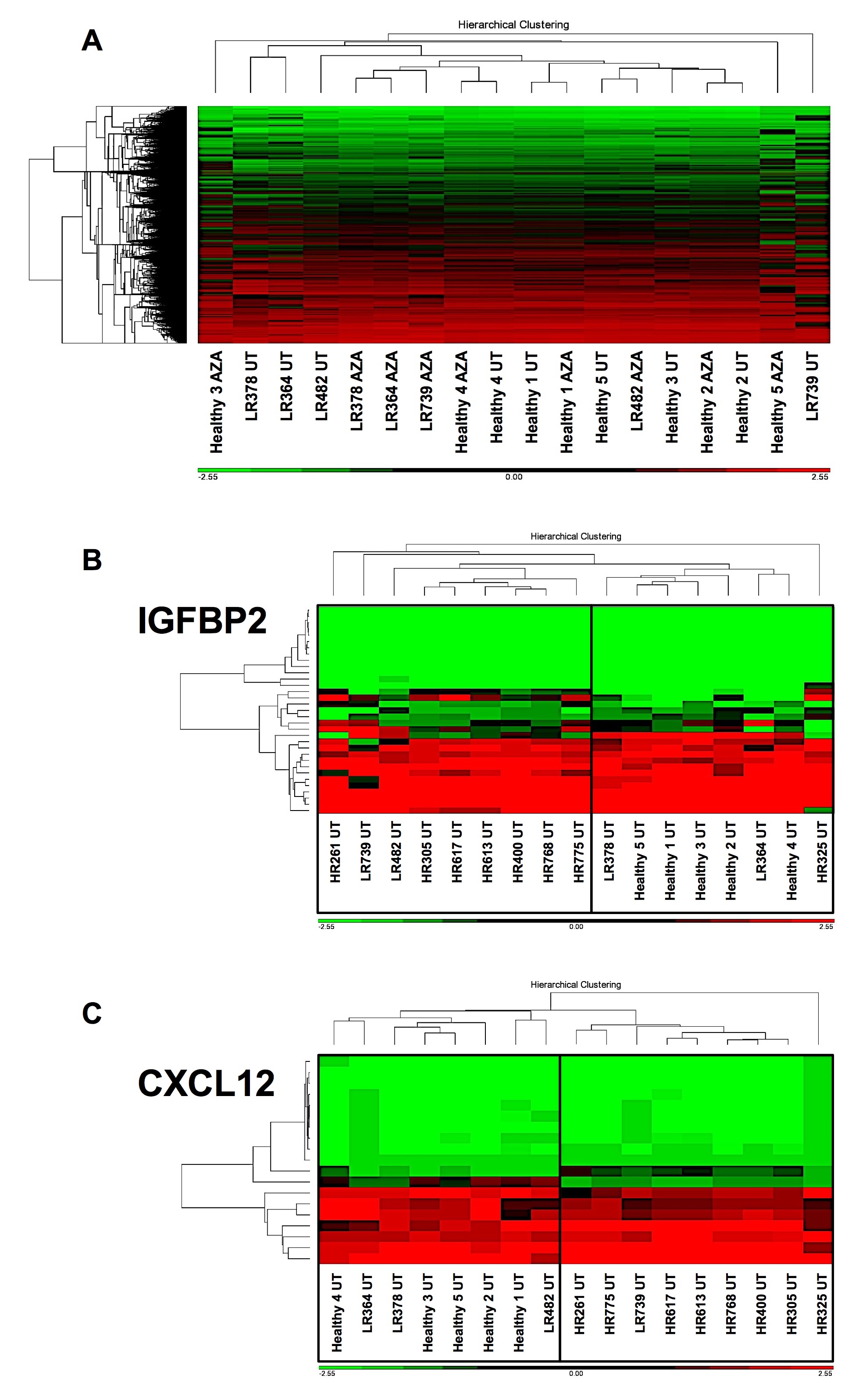


**Supplementary Figure 4.** **A)** Hierarchical clustering of samples using CpG loci that are significantly differentially methylated is not as effective for LR-MDS-MSCs vs healthy MSCs. B and C) Hierarchical clustering of samples for IGFBP2 and CXCL12, respectively. qPCR of these genes are given in Figure 1.


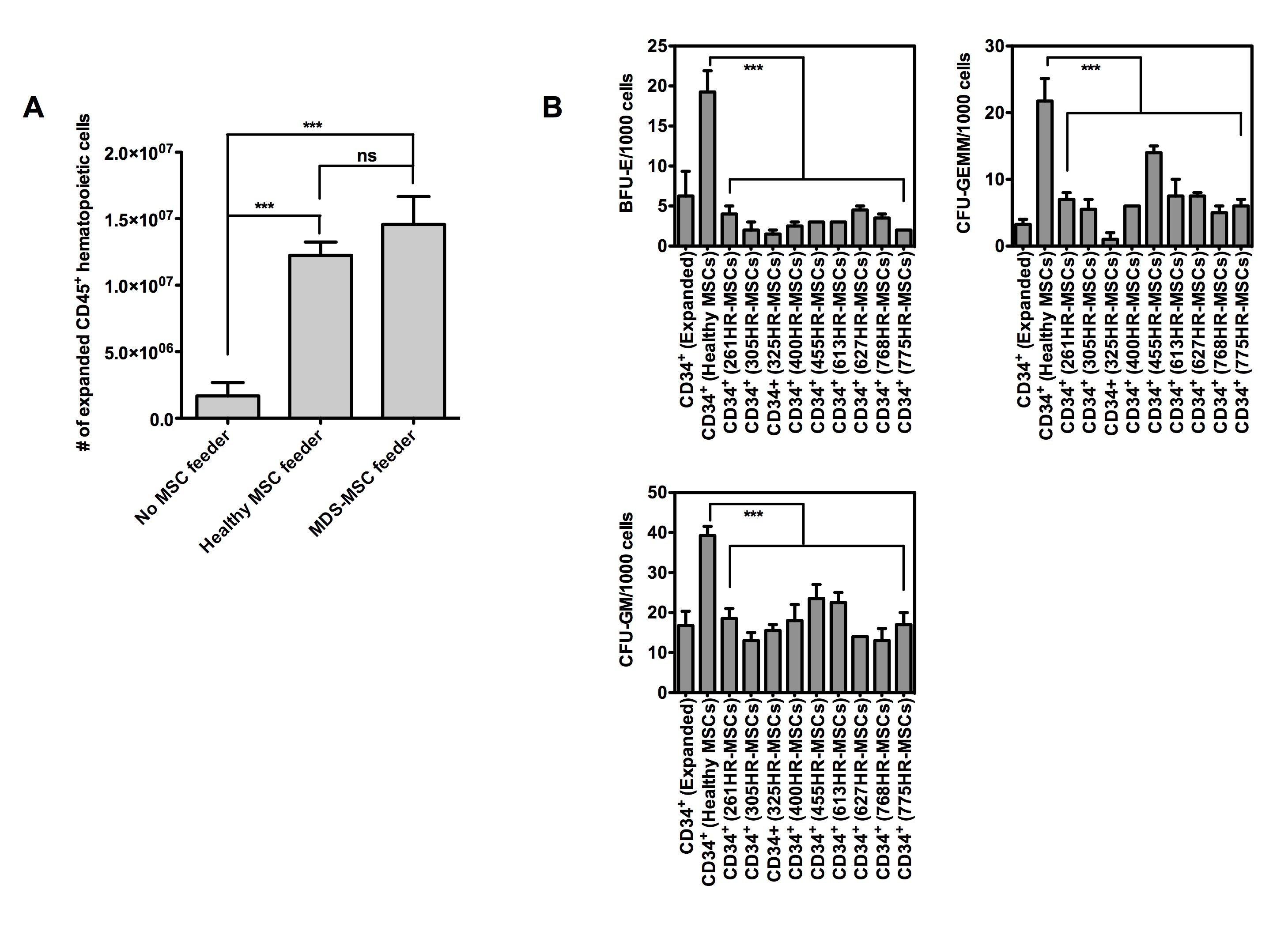


**Supplementary Figure 5. Expanded healthy HSPCs in different co-culture systems. A)** Co-culture with healthy (n = 6) or MDS-MSCs (n = 16) resulted in comparable numbers of CD45^+^ hematopoietic cells (1.22 ± 0.1 × 10^7^ and 1.47 ± 0.23 × 10^7^ cells, respectively; *p* = 0.163); and these expansion numbers were significantly greater (*p* < 0.001) compared to cultures without MSC feeders (1.68 ± 1.01 × 10^6^ cells, n = 6). **B)** BFU-E, CFU-GEMM and CFU-GM counts for each HR-MDS sample setup shown in Figure 3C. **p* < 0.05, ***p* < 0.01, ****p* < 0.001, ns = Not significant. Non-paired student’s t-test was performed. All data represented as mean ± SEM.


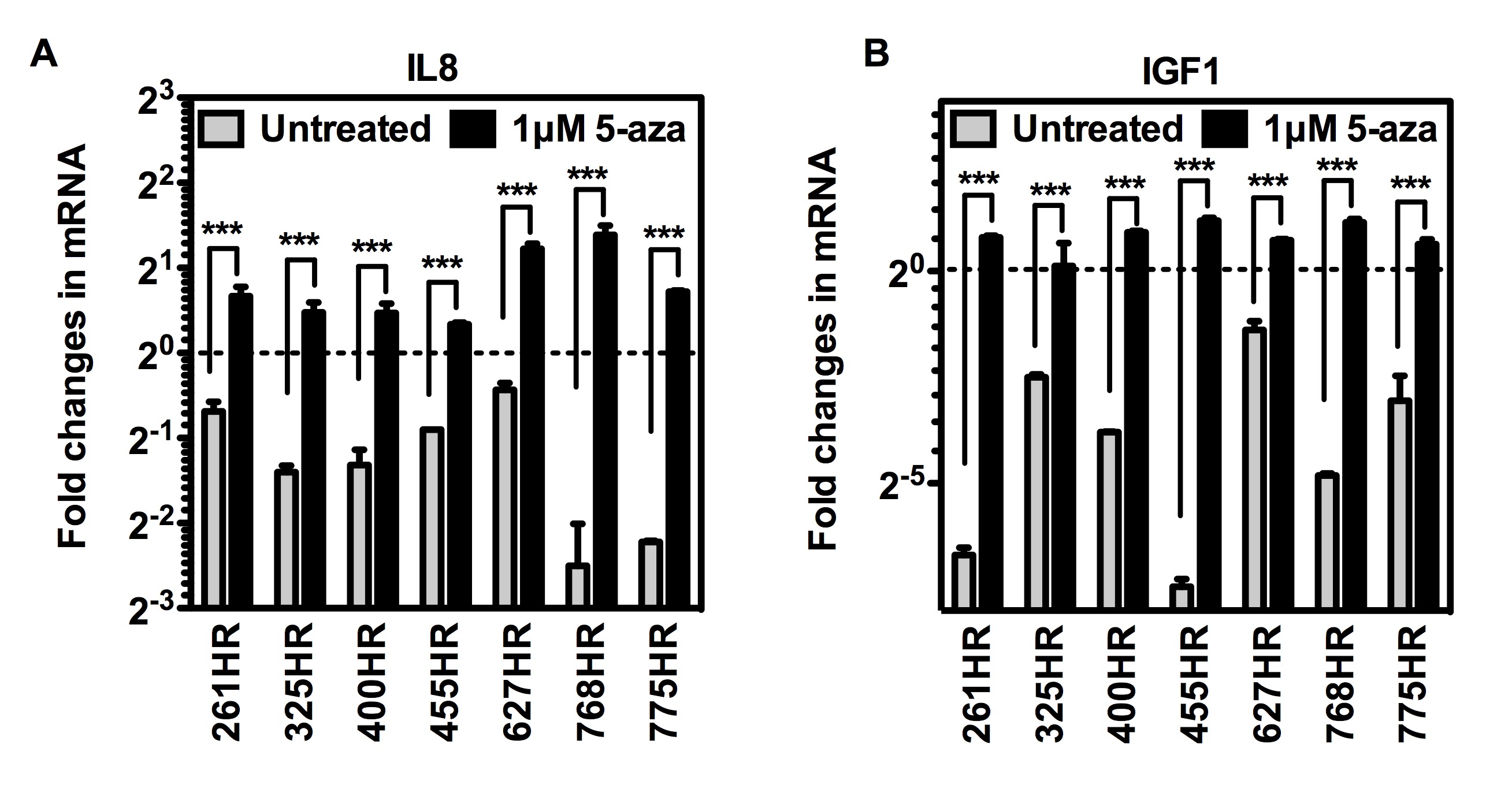


**Supplementary Figure 6. A and B)** qPCR analysis of MDS-MSCs (n = 7, P1) for expression of IL8 and IGF1 before and after 5-aza treatments. Representative data normalized to a healthy control. After treatment, gene expression of these genes was significantly increased. Similar trends were observed with Osterix and CXCL12 gene expression (Figures 3F and G), suggesting a restoration of normal MSC phenotypic function.


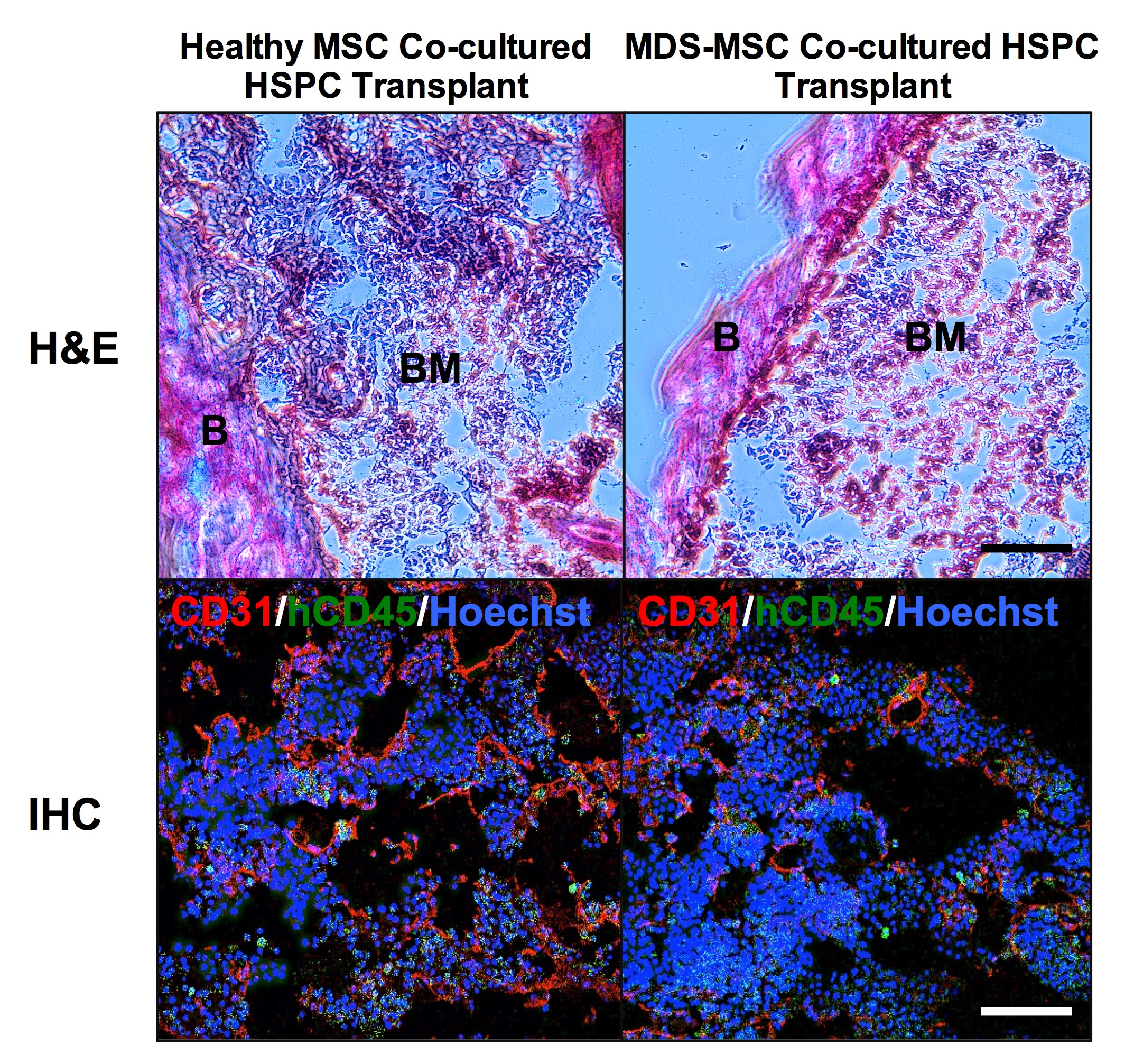


**Supplementary Figure 7:** Representative histological images of femoral bone marrow 8 weeks after primary transplantation with CD34^+^ HSPCs co-cultured with healthy vs MDS-MSCs *in vitro* (Figure 5A). Both H&E as well as immunohistochemical staining are shown. We did not observe any significant differences in the general marrow architecture (vascularity and cellularity) between those engrafted with healthy-MSC or MDS-MSC co-cultured HSPCs. Fibrosis and hypercellularity were also not evident in our marrow samples. BM = bone marrow, B = bone. Scale bar = 200 μm.


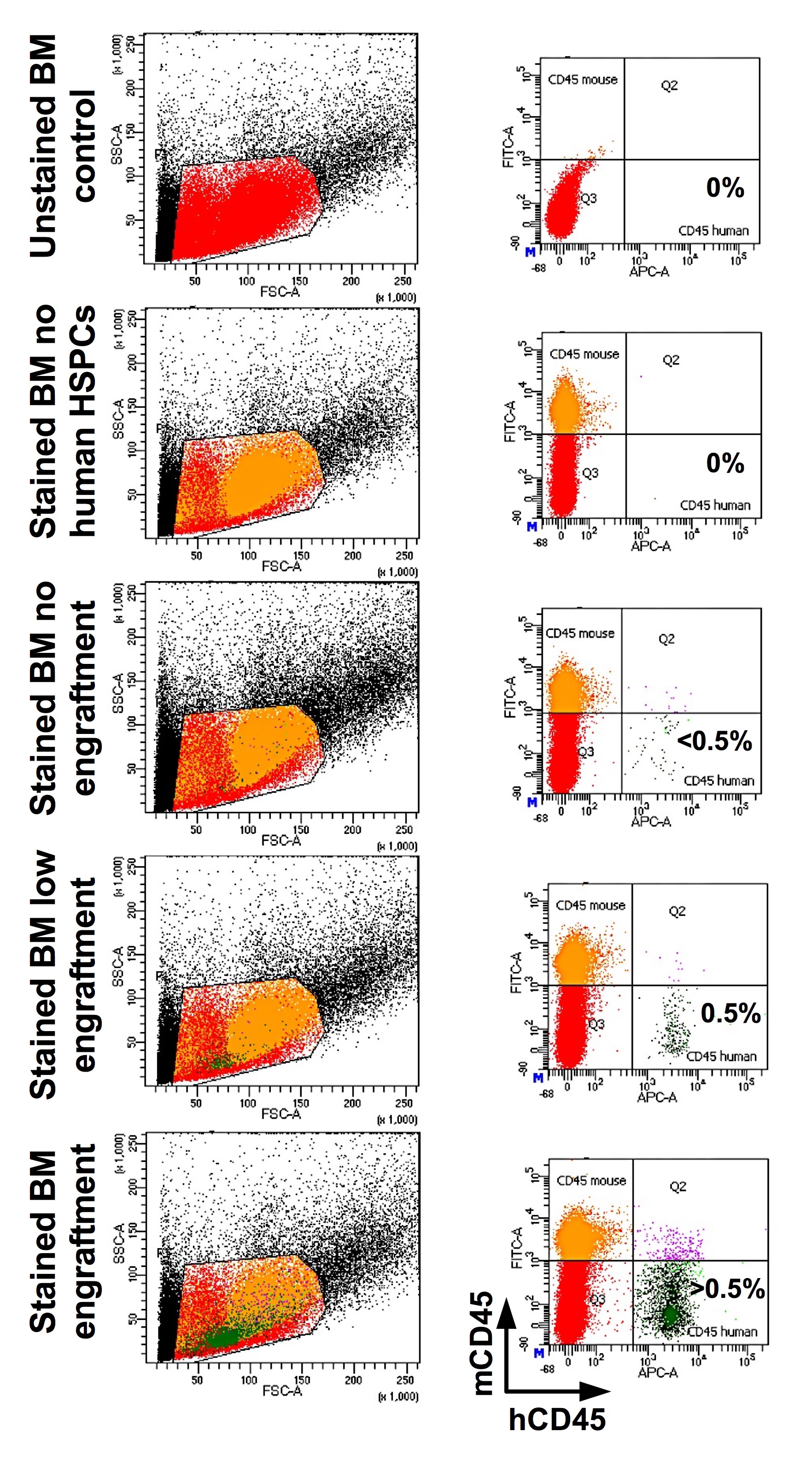


**Supplementary Figure 8:** Representative flow cytometry plots used to determine chimerism and engraftment in the bone marrow. The detection threshold of 0.5 % is arbitarily set based on flow cytometry plots captured of bone marrow cells with varying degrees of engrafted human cells. These plots show a detection limit of ~0.1 - 0.5 % for hCD45 cells and we have used 0.5 % as a more stringent cut off for engraftment.

**
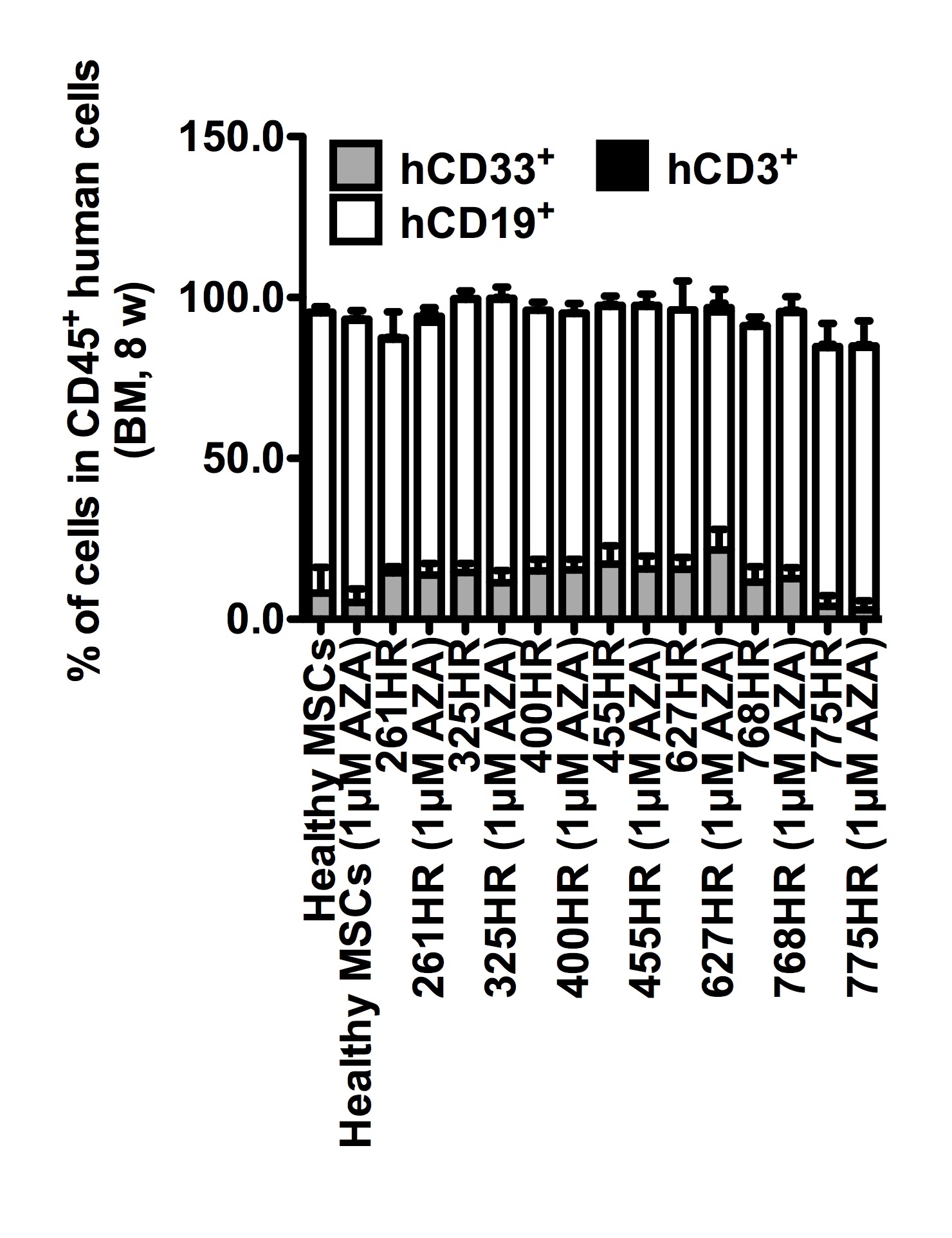
**

**Supplementary Figure 9.** Multilineage differentiation of donor CD34^+^ HSPCs in the BM of primary transplanted recepient mice. Frequencies of human donor myeloid (CD45^+^CD33^+^), T-cell (CD45^+^CD3^+^) and B-cell (CD45^+^CD19^+^) found in femoral BM 8 weeks after transplantation (n = 5 mice). These results show no difference in multilineage differentiation capacity after exposure to MDS stroma.

**Supplementary Table 1.** Primers used for qPCR.


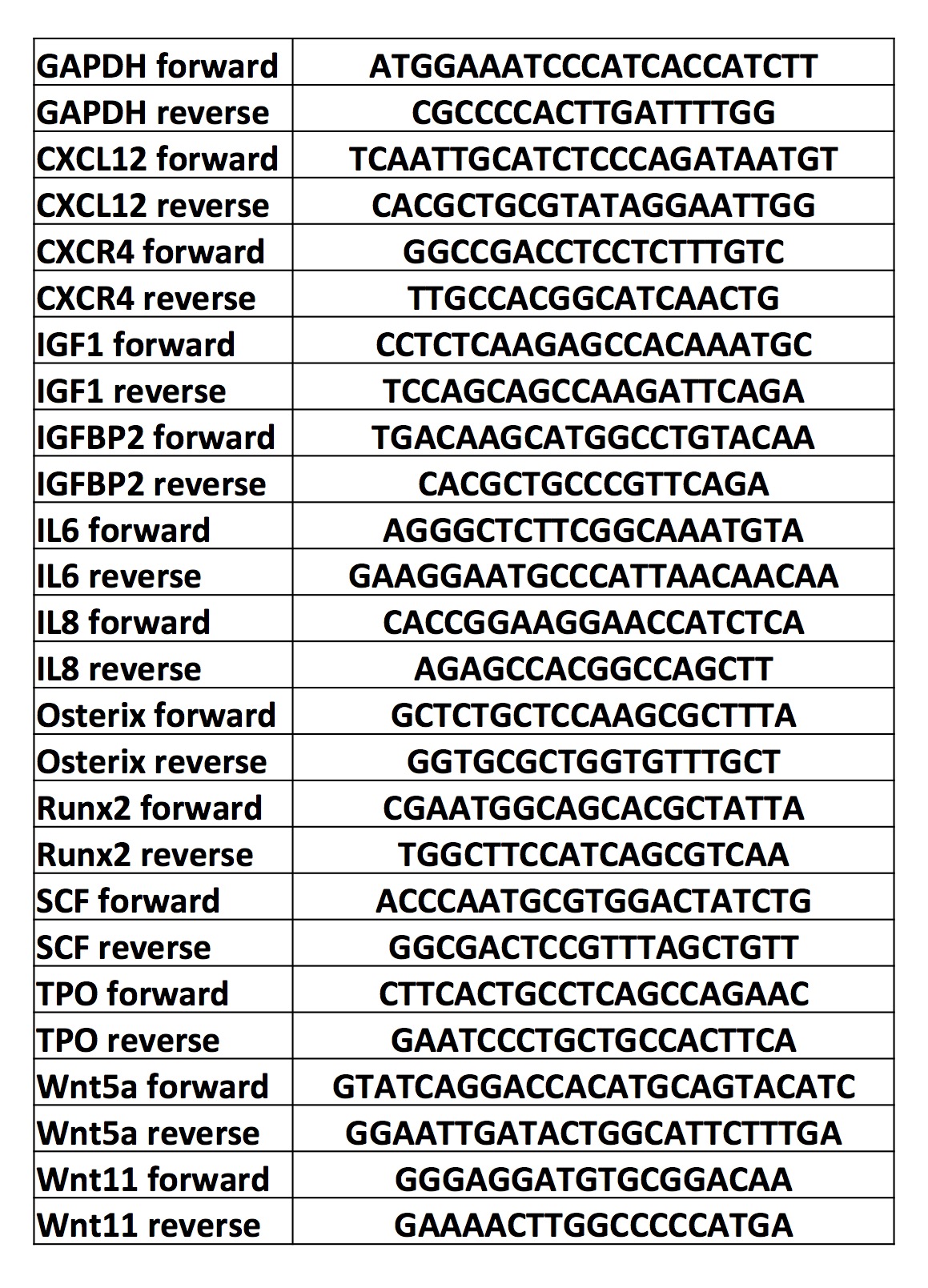


**References**

1 Xing, Y. *et al.* An expectation-maximization algorithm for probabilistic reconstructions of full-length isoforms from splice graphs. *Nucleic Acids Res* **34**, 3150-3160, doi:10.1093/nar/gkl396 (2006).

2 Law, C. W., Chen, Y., Shi, W. & Smyth, G. K. voom: Precision weights unlock linear model analysis tools for RNA-seq read counts. *Genome Biol* **15**, R29, doi:10.1186/gb-2014-15-2-r29 (2014).
